# Jiedu Fuzheng decoction improves the proliferation, migration, invasion and EMT of non-small cell lung cancer via the Wnt/β-catenin pathway

**DOI:** 10.1186/s13008-023-00105-7

**Published:** 2023-12-16

**Authors:** Fang Fang, Xiaowei Jin, Jinming Meng, Jiaqi He, Jiaxiao Wang, Changhong Wang, Sheng Xie, Wei Shi

**Affiliations:** 1 The Second Ward of Oncology, The First Affiliated Hospital of Guangxi University of Chinese Medicine, No. 327, Xianhu Avenue, Qingxiu District, Nanning, 530001 Guangxi Zhuang Autonomous Region People’s Republic of China; 2 Department of Traditional Chinese Medicine, Yunnan Cancer Hospital, Kunming, 650018 Yunnan People’s Republic of China; 3 Guangxi University of Chinese Medicine, Nanning, 530001 Guangxi Zhuang Autonomous Region People’s Republic of China; 4 Preventive Treatment of Disease Center, The First Affiliated Hospital of Guangxi University of Chinese Medicine, No. 89-9, Dongge Road, Qingxiu District, Nanning, 530001 Guangxi Zhuang Autonomous Region People’s Republic of China

**Keywords:** Lung cancer, Jiedu Fuzheng decoction, Wnt/β-catenin, Migration, Invasion

## Abstract

**Objectives:**

This study aimed to investigate the effect of Jiedu Fuzheng decoction (JFD) in non-small cell lung cancer (NSCLC) and its potential therapeutic mechanism.

**Results:**

We prepared JFD-medicated serum from rats and treated NSCLC cells (A549 and NCI-H1650) with 0.5, 1, and 2 mg/mL JFD-medicated serum. CCK-8 and colony formation assays were used to detect cell proliferation. Transwell assays showed that JFD attenuated cell migration and invasion. JFD and SKL2001 (Wnt/β-catenin activator) were simultaneously used to treat NSCLC cells to verify that JFD regulated the biological behavior of NSCLC via Wnt/β-catenin signaling. It was found that 2 mg/mL JFD had the most significant effect on the activity of NSCLC cells. JFD attenuated proliferation and metastasis but increased the proportion of apoptotic cells. At the same time, JFD downregulated N-cadherin, vimentin and β-catenin protein expression in cancer cells. SKL2001 could restore the improvement of JFD on proliferation, metastasis and apoptosis.

**Conclusion:**

This study confirmed that JFD suppressed the occurrence and development of NSCLC by regulating Wnt/β-catenin signaling and provided a novel therapeutic scheme for NSCLC.

## Highlights

1. JFD inhibited the activity and proliferation of NSCLC cells.
2. JFD inhibited the migration and invasion of NSCLC cells.
3. Activation of the Wnt/β-catenin pathway partially restored the influence of JFD on the NSCLC cell malignant phenotype.

## Background

Lung cancer is recognized as the most common malignant tumor, of which 85% is non-small cell lung cancer (NSCLC) [1, 2]. Although there have been encouraging results in the treatment of NSCLC patients through surgical resection, radiotherapy, chemotherapy, immunotherapy and targeted therapy, the prognosis of NSCLC remains poor [3]. Lung cancer kills more than a million people a year, and a significant amount of time and money need to be invested in therapeutic studies for NSCLC each year [4]. Therefore, it is still relevant to explore a novel treatment scheme for NSCLC.

In Asia, especially in China, Chinese herbs have been used as antitumor drugs in clinical treatment. Traditional Chinese medicine formulations not only play a significant role in ameliorating cancer development but also reduce adverse reactions and complications caused by chemotherapy or radiotherapy [2]. Jiedu Fuzheng decoction (JFD) is a Chinese herb that is mainly composed of 13 drugs, such as *Jieducao*, *Nervilia fordii*, *Chrysanthemum indicum*, *Oldenlandia*, *Astragalus membranaceus* and *Atractylodes macrocephala* [5]. The study of Yang et al. confirmed that JFD treatment could reduce the mortality of patients with hepatocarcinoma and extend their lifespan [6]. Studies of ovarian cancer (OV) have also found that FJD may increase the sensitivity of OV to cisplatin by mediating the phosphoinositide 3-kinase (PI3K)/AKT or NF-κB pathways [7]. These studies suggest that JFD has a potential role in improving cancer. Our previous experiments confirmed that JFD could reduce proliferation, but whether JFD can affect NSCLC metastasis has not been reported.

The abnormal proliferation and metastasis of tumor cells are mostly affected by carcinogenic signaling, such as Notch, Wnt/β-catenin, MAPK and Hippo axi [8]. A variety of cellular biological behavior processes are mediated by Wnt/β-catenin signaling. The disorder of the Wnt/β-catenin cascade causes malignant tumors to occur and develop [9]. At the same time, Wnt/β-catenin signaling is regulated by multiple traditional Chinese medicines. Astragalus polysaccharides can induce apoptosis of gastric cancer cells by mediating the Wnt/β-catenin axis, causing cell cycle arrest [10]. Chinese herbal extracts can prevent the progression of SW403 cells by attenuating the Wnt/β-catenin pathway [11]. It is not clear whether JFD can improve the occurrence and progression of NSCLC via Wnt/β-catenin signaling.

In this research, JFD-containing serum was prepared, and NSCLC cells were treated with JFD to verify the influence of JFD on cancer cell behavior. Furthermore, a Wnt/β-catenin activator was added to verify that JFD regulates the development of NSCLC via the Wnt/β-catenin axis. Our study proposes a new therapeutic strategy for therapeutic schemes of NSCLC.

## Results

### JFD suppresses the proliferation of NSCLC cells

**Fig. 1:**
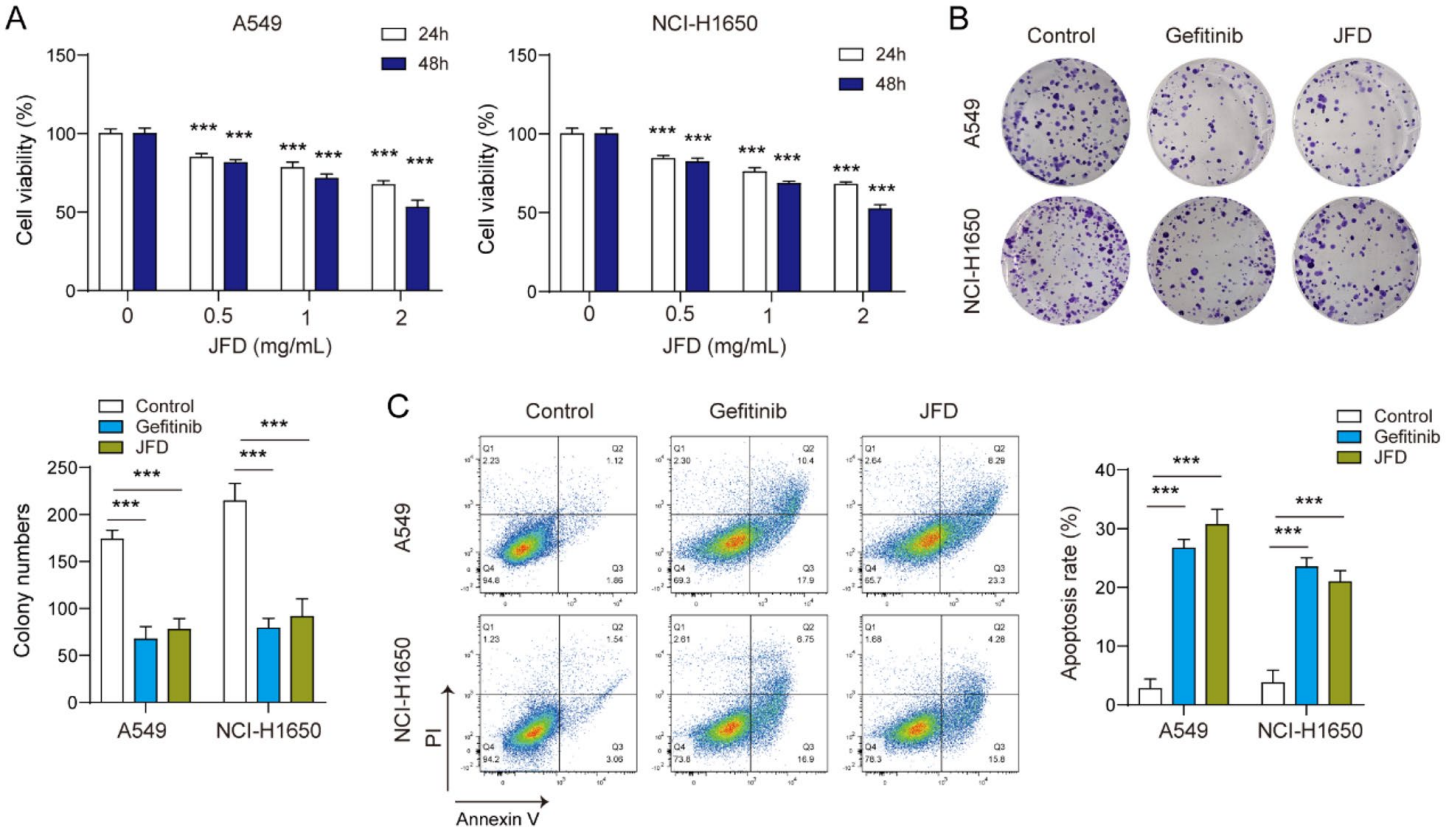
JFD inhibits the proliferation of NSCLC cells. **A** CCK-8 was used to detect cell viability. **B** Colony formation was used to determine cell proliferation. **C** The level of apoptosis was detected by flow cytometry. n = 3. ***p < 0.001

We evaluated the impact of JFD on proliferation and apoptosis. JFD reduced the viability of cancer cells, and 2 mg/mL JFD treatment for 48 h had the best effect (Fig. 1A). JFD significantly reduced the proliferation of tumor cells (Fig. 1B). Furthermore, JFD remarkably promoted apoptosis (Fig. 1C). In summary, JFD can suppress the proliferation of cancer cells

### JFD suppresses metastasis of lung cancer cells

**Fig. 2:**
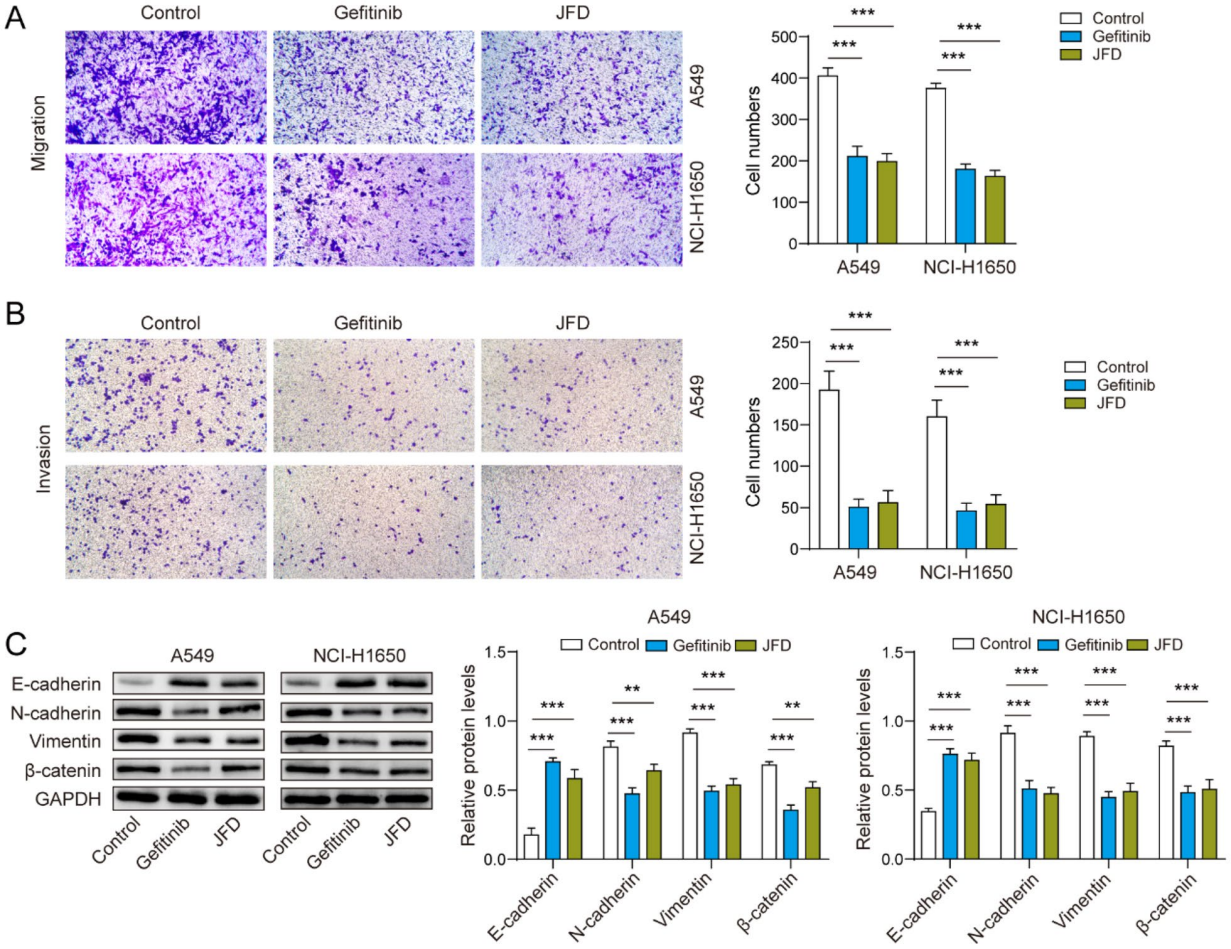
JFD represses the migration and invasion of NSCLC cells. **A** Transwell assays were used to detect cell migration. **B** Transwell assays were used to detect cell invasion. **C** The protein expression levels of N-cadherin, E-cadherin, Vimentin and β-catenin were detected by western blot. n = 3. **p < 0.01, ***p < 0.001

Next, we examined the effects of JFD on the metastasis of A549 and NCI-H1650 cells. Similarly, JFD markedly reduced the migration (Fig. 2A) and invasion (Fig. 2B) of cancer cells. Epithelial–mesenchymal transition (EMT) marker protein (N-cadherin, E-cadherin, Vimentin and β-catenin) expression mediated by JFD was further analyzed. JFD significantly upregulated E-cadherin but downregulated N-cadherin, vimentin and β-catenin (Fig. 2C). In conclusion, JFD inhibits the metastasis of lung cancer cells.

### JFD exerted suppressive roles in the proliferation of NSCLC cells via the Wnt/β-catenin axis

**Fig. 3:**
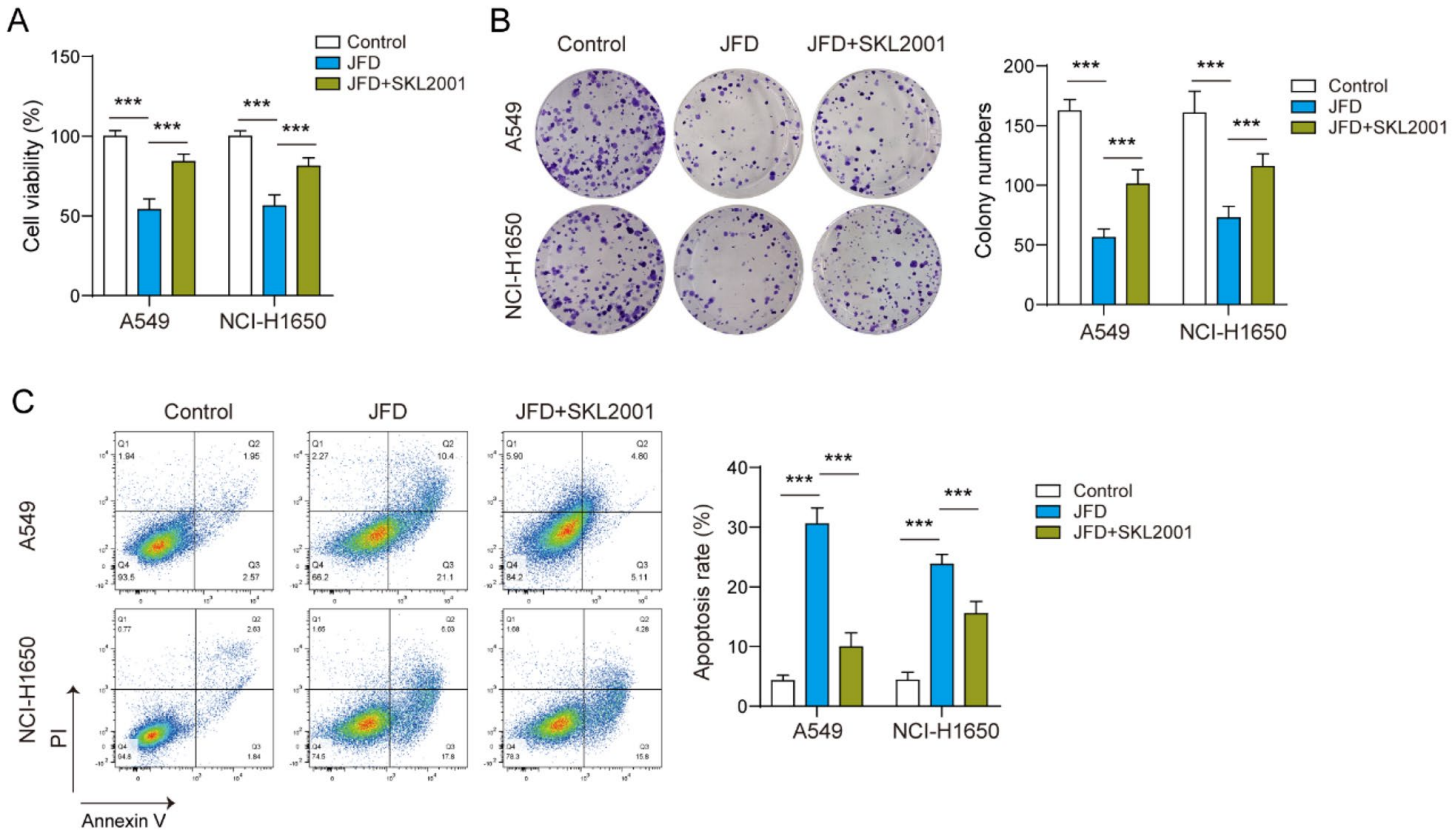
JFD inhibits the proliferation of NSCLC cells through the Wnt/β-catenin pathway. **A** Cell viability was determined by CCK-8. **B** Cell proliferation was determined according to colony formation. **C** Apoptosis was detected by flow cytometry. n = 3. ***p < 0.001

The change in Wnt/β-catenin axis activity is related to the proliferation and metastasis of cancer [12]. The level of β-catenin protein was downregulated after JFD treatment (Fig. 2C). To further explore whether JFD regulates cell proliferation via the Wnt/β-catenin axis, we cotreated NSCLC cells with SKL2001 and JFD. We observed that stimulating the Wnt/β-catenin axis partially restored the influences of JFD on cell viability (Fig. 3A) and proliferation (Fig. 3B). Consistently, JFD-induced apoptosis was reduced by activating Wnt/β-catenin signaling (Fig. 3C). The above results indicate that JFD may reduce the proliferation of NSCLC cells via the Wnt/β-catenin axis.

### JFD inhibits metastasis of cells via the Wnt/β-catenin axis

**Fig. 4:**
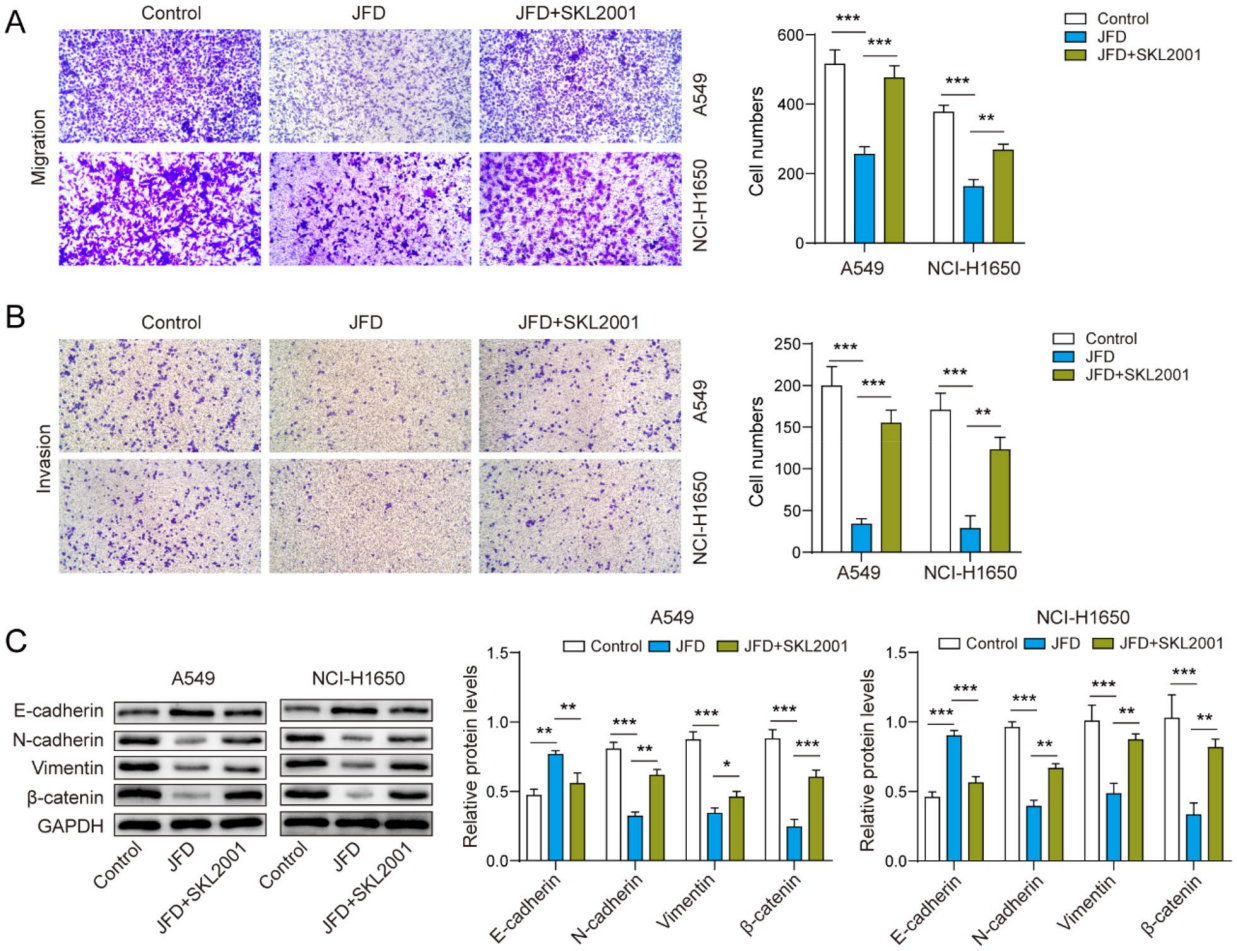
JFD suppresses the migration and invasion of NSCLC cells through the Wnt/β-catenin pathway. **A** Cell migration was detected by Transwell assays. **B** Transwell assays were used to detect cell invasion. **C** The protein expression levels of N-cadherin, E-cadherin, Vimentin and β-catenin were detected by western blot. n = 3. *p < 0.05, **p < 0.01, ***p < 0.001

Finally, we evaluated the regulatory effect of JFD on the metastasis of NSCLC via the Wnt/β-catenin axis. By using JFD and SKL2001 to treat cancer cells simultaneously, it was found that stimulating the Wnt/β-catenin axis restored the inhibitory effect of JFD on cell migration (Fig. 4A) and invasion (Fig. 4B). Similarly, JFD inhibited the process of EMT, while stimulating the Wnt/β-catenin pathway downregulated E-cadherin and upregulated N-cadherin, vimentin and β-catenin (Fig. 4C). In general, JFD may suppress the metastasis of NSCLC via the Wnt/β-catenin axis.

## Discussion

**Fig. 5:**
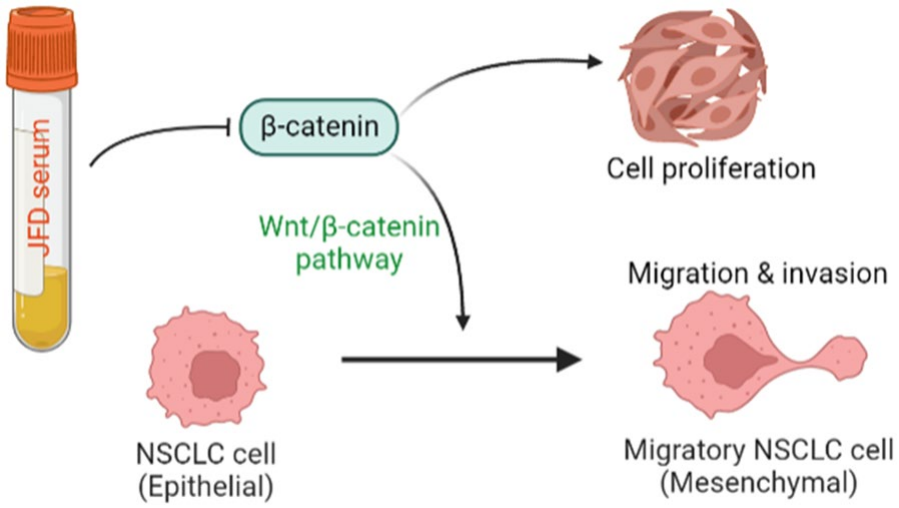
JFD inhibits the proliferation, migration and invasion of NSCLC cells by suppressing the Wnt/β-catenin pathway (by FigDraw)

At present, the incidence and mortality of primary lung cancer in China still rank first [13]. Although the current treatment of NSCLC patients has yielded encouraging results, the recurrence rate of patients after surgical resection is still high [14]. An increasing number of traditional Chinese medicine prescriptions, which have few adverse reactions and a low cost, are used to treat various diseases, including cancer [15]. Our study confirmed that NSCLC cell proliferation and metastasis could be improved by JFD. Further exploration of its molecular mechanism revealed that JFD suppressed the development of lung cancer by inhibiting the Wnt/β-catenin axis (Fig. 5). Our study contributes a reliable research basis for the treatment of cancer in clinical practice, especially NSCLC.

As an effective adjuvant therapy, traditional Chinese medicine has been applied in the clinical treatment of lung cancer. Traditional Chinese medicine can enhance the effect of chemotherapy and radiotherapy through synergistic effects and reduce the adverse reactions and toxicity to patients [16]. JFD has been shown to improve many types of cancer. JFD can extend the lifetime of mice with liver cancer and reduce the metastasis of cancer [17]. A recent study showed that JFD improves the efficacy of cisplatin therapy in ovarian cancer and activates NF-κB signaling by suppressing the PI3K/AKT/mTOR axis [7]. Our study also reached a similar conclusion. We treated cancer cells with different concentrations of JFD and found that JFD inhibited the activity of cancer cells in a concentration-dependent manner. Further detection also confirmed that JFD could regulate the proliferation, migration, invasion and EMT of cancer cells. The inhibitory effect of traditional Chinese medicine decoction on tumors may be achieved through the synergistic effect of multiple targets and multiple signaling pathways [18]. At present, there are many studies and applications of JFD in the treatment of liver cancer. JFD can reduce the expression of genes and related pathways of TP53, CCND1, p-EGFR, EGF, VEGFA, JUN, IL-6, COX-2, AKT1 and MAPK1 in liver cancer cells [19]. We found that JFD inhibited the activation of the Wnt/β-catenin pathway in NSCLC cells. Our study first verified the therapeutic effect of JFD in NSCLC.

The Wnt/β-catenin signaling pathway affects cell growth, development, evolution, and homeostasis [20]. The transcriptional activity of the oncogene β-catenin is essential for regulating the development of cancer [21]. The Wnt/β-catenin axis is crucial in NSCLC cell lines. The activation of the Wnt/β-catenin axis by promoting the level of β-catenin in cancer cells can promote the development of NSCLC [22]. Upregulation of β-catenin protein level in NSCLC may increase the interaction between β-catenin and E-cadherin and improve the prognosis of NSCLC [23]. In previous reports, the main active substances of JFD, such as *Scutellaria baicalensis*, *Astragalus membranaceus* and *Taraxacum officinale*, were related to stimulating Wnt/β-catenin signaling [24–26]. We found increased β-catenin in JFD-treated NSCLC cells. Further treatment of cells treated with JFD by using SKL2001 reversed the improvement of JFD on cancer cell proliferation and metastasis. In addition, we also observed that JFD significantly increased E-cadherin protein and decreased N-cadherin and vimentin protein levels in lung cancer cells. The changes in the protein levels of these markers indicate that JFD can lead to the transformation of mesenchymal cells into epithelial cells. Similarly, SKL2001 can restore JFD’s suppression of EMT transition. Therefore, we speculate that JFD can mediate the development of NSCLC by the Wnt/β-catenin axis. EMT is an important process of cancer metastasis, drug resistance and cancer stem cells [27]. We will continue to explore the influence of JFD on lung cancer drug resistance, radiotherapy and chemotherapy.

In conclusion, our study confirmed that JFD regulated the behavior of NSCLC. At the same time, it was found at the molecular level that JFD affected the development of cancer cells through the Wnt/β-catenin axis. However, our research also has certain limitations, and we have only confirmed our conclusions at the cellular level. The use of animal models to study cancer metastasis can better support our conclusions, and we will next verify our conclusions at the animal level.

## Methods

### JFD preparation

The main components of the decoction were *Jieducao* 15 g, *Forsythia suspensa* 15 g, *Nervilia fordii* 15 g, *Chrysanthemum indicum* 15 g, *Scutellaria baicalensis* 15 g, *Chinese violet* 10 g, *Taraxacum officinale* 30 g, *Oldenlandia* 20 g, *Sculellaria barbata* 20 g, *Raw oyster* 30 g, *Pericarpium Citri Reticulatae* 15 g, *Astragalus membranaceus* 30 g, and *Atractylodes macrocephala* 20 g. In the first decoction, medicines were soaked with 1200 mL water for 30 min and decocted for 90 min to obtain approximately 200 mL of the medicinal liquid. In the second decoction, 900 mL water was added, and the decoction was carried out for 90 min to obtain approximately 225 mL of liquid. The first and second decoctions were mixed and concentrated to the desired concentration. The low, medium and high doses of JFD were 0.5 mg, 1.0 mg and 2.0 mg crude drug per 1 mL, respectively. JFD was stored at 4 °C for subsequent experiments.

### Preparation of medicated serum

Fifteen healthy SPF SD rats (6–8 weeks, 140–180 g) were purchased from Guangdong Medical Experimental Animal Center (Guangdong, China). The rats were randomized into 5 groups (3 rats/group): the control group, gefitinib group (10 µM), JFD low-dose group (0.5 mg/mL), JFD middle-dose group (1 mg/mL) and JFD high-dose group (2 mg/mL). Control rats were given 9% NaCl every day; the gefitinib group was given gefitinib solution by gavage; and the rats in the JFD groups were given low, medium and high doses of JFD decoction by gavage. Gavage was performed once in the morning and evening, 2 mL each time, for 5 consecutive days. The rats were fasted at night before blood collection, and blood samples were drawn from the abdominal aorta 1 h after the last administration on the next day. After standing for 2 h, the samples were centrifuged for 8 min (3000 rpm). Then, the collected serum was incubated at a constant temperature (56 °C) for 30 min, and the inactivated complement was sterilized. Finally, medicated sera were stored at − 20 °C. All animal experiments were approved by the Ethics Committee of The First Affiliated Hospital of Guangxi University of Chinese Medicine.

### Cell culture and treatments

Human NSCLC cell lines (A549 and NCI-H1650) were obtained from the American Type Culture Collection (TCGA, Manassas, Virginia, USA). Cells were cultured in DMEM (Sigma‒Aldrich, Darmstadt, Germany) containing 10% fetal bovine serum (FBS) and 1% penicillin/streptomycin (Gibco, Grand Island, New York, USA) in a 5% CO_2_ incubator at 37 °C. The cells were cultured in medium containing drug-containing serum. For β-catenin agonist treatment, NSCLC cells were treated with 30 µM SKL2001 (MedChem Express, Monmouth Junction, NJ, USA) for 24 h.

### Cell counting Kit-8 (CCK-8) assay

Cells to be detected were inoculated into 96 wells at 5 × 10^3^ cells/well and incubated for 0, 24, and 48 h. Subsequently, 10 µL of CCK-8 solution (Abcam, Cambridge, MA, USA) was added to each well and incubated at 37 °C for 4 h. Finally, the value at 450 nm was read by a microplate reader (Varioskan LUX, Thermo Scientific, Massachusetts, USA).

### Colony formation assay

A549 and NCI-H1650 cells were inoculated into 6-well plates (2 × 10^3^ cells/well). After 14 days, the cells were fixed with 4% paraformaldehyde and stained with 0.1% crystal violet (Beyotime, Shanghai, China) for 30 min. Colony images were taken with a camera, and aerobic colonies were counted using ImageJ software.

### Cell apoptosis assay

The cell suspensions were mixed with 1 Annexin V-fluorescein isothiocyanate (10 µL) and propidium iodide (PI, 5 µL) for 30 min at 25 °C in the dark. The apoptosis ratio was determined by FACSCalibur flow cytometry.

### Transwell migration and invasion assays

Cells to be detected (2 × 10^4^ cells) without serum were inoculated in the upper chamber of a Transwell (Corning, New York, USA). Normal culture medium containing serum was placed in the lower chamber. For the detection of cell invasion, Matrigel (Sigma-Aldrich) was used to cover the Transwell insert. After 24 h, the cells that did not migrate to or invade the upper chamber were removed. Transwell lower chamber cells were fixed and stained with methanol and crystal violet. Finally, five fields of view were randomly selected using a microscope for observation and counting.

### Western blot

A549 and NCI-H1650 cells were placed on ice and lysed with RIPA buffer for 40 min. Total protein levels were quantified. The protein (20 µg) was transferred to a PVDF membrane after electrophoretic separation and blocked with 5% skim milk for 1 h, followed by overnight incubation of the primary antibody with the membrane at 4 °C. The next day, the sample was incubated with HRP-conjugated antibody at room temperature for 30 min. Finally, enhanced chemiluminescence reagents (KeyGen, Nanjing, China) were used for development and recording. The following primary antibodies were purchased from Abcam: anti-N-cadherin (ab76011, 1:5000), anti-E-cadherin (ab40772, 1:10,000), anti-Vimentin (ab92547, 1:2000), anti-β-catenin (ab32572, 1:5000), and anti-GAPDH (ab9485, 1:2500).

### Statistical analysis

All data are expressed as the mean ± standard deviation (SD) of three experiments. Statistical analysis was performed using GraphPad Prism 8.0 software. The comparison among multiple groups was assessed by one-way analysis of variance (ANOVA), followed by Tukey’s post hoc test. *P* < 0.05 was considered statistically significant.

## Data Availability

The datasets used or analyzed during the current study are available from the corresponding author upon reasonable request.

## Abbreviations

NSCLC: Non-small cell lung cancer
OV: Ovarian cancer
JFD: Jiedu Fuzheng decoction
EMT: Epithelial–mesenchymal transition
PI3K: Phosphoinositide 3-kinase
FBS: Fetal bovine serum
CCK-8: Cell counting kit-8
SD: Standard deviation
